# Association of Total Bilirubin With All-Cause and Cardiovascular Mortality in the General Population

**DOI:** 10.3389/fcvm.2021.670768

**Published:** 2021-06-18

**Authors:** Ziwei Chen, Jing He, Chu Chen, Qi Lu

**Affiliations:** ^1^Department of Cardiology, Affiliated Hospital of Nantong University, Nantong, China; ^2^Department of Oncology, Affiliated Hospital of Nantong University, Nantong, China

**Keywords:** total bilirubin, oxidative stress, all-cause mortality, cause-specific mortality, National Health and Nutrition Examination Survey

## Abstract

**Objective:** The study aims to investigate the association of total bilirubin with all-cause and cause-specific mortality in the general population.

**Methods:** A total of 37,234 adults from the United States National Health and Nutrition Examination Survey 1999–2014 were enrolled. Baseline levels of total bilirubin associated with risk of mortality were evaluated on a continuous scale (restricted cubic splines) and by quartile categories with Cox regression models.

**Results:** Higher levels of total bilirubin was positively associated with an increased risk of all-cause mortality [hazard ratio (HR) 1.59, 95% confidence interval (CI) 1.46–1.72; *p* < 0.001]. Compared with individuals with the lowest quartile of bilirubin, the multivariable adjusted hazard ratio for all-cause mortality was 1.25 (1.14–1.37) for individuals in the highest quartile. Restricted cubic splines indicated that the association was non-linear in cardiovascular mortality and cancer mortality while linear in all-cause mortality.

**Conclusions:** Total bilirubin was associated with all-cause and cause-specific mortality in the general population.

## Introduction

As an end product of heme degradation, bilirubin has antioxidant and anti-inflammatory effects (1). Animal experiments showed that bilirubin improved vascular dysfunction in atherosclerosis and myocardial infarction (2). Many studies have confirmed an inverse association of plasma bilirubin levels with the prognosis of many diseases, such as diabetes (3, 4), arteriosclerotic cardiovascular disease (5), and hypertension (6). The protective function can be explained by the anti-oxidative, anti-inflammatory, and anti-adipogenic roles of bilirubin, making bilirubin a potential therapeutic target (7).

However, studies on the association between bilirubin levels and the risk of mortality have provided conflicting results. Previous studies have suggested bilirubin to be a protective marker in cardiovascular diseases (8, 9) and cancer (10), showing an inverse relationship. Some studies reported that increased bilirubin was a risk factor of mortality in patients with ischemic heart failure (11), stroke (12), or traumatic brain injury patients (13). In addition, total bilirubin levels were found to be associated with cardiovascular mortality in an L-shaped or U-shaped relationship (14, 15).

Moreover, there was a lack of studies on the association between total bilirubin levels and all-cause mortality in the general population. Therefore, we examined the association of total bilirubin levels with all-cause and cause-specific mortality. We also investigated whether such associations were linear.

## Methods

### Study Population

**Figure 1:**
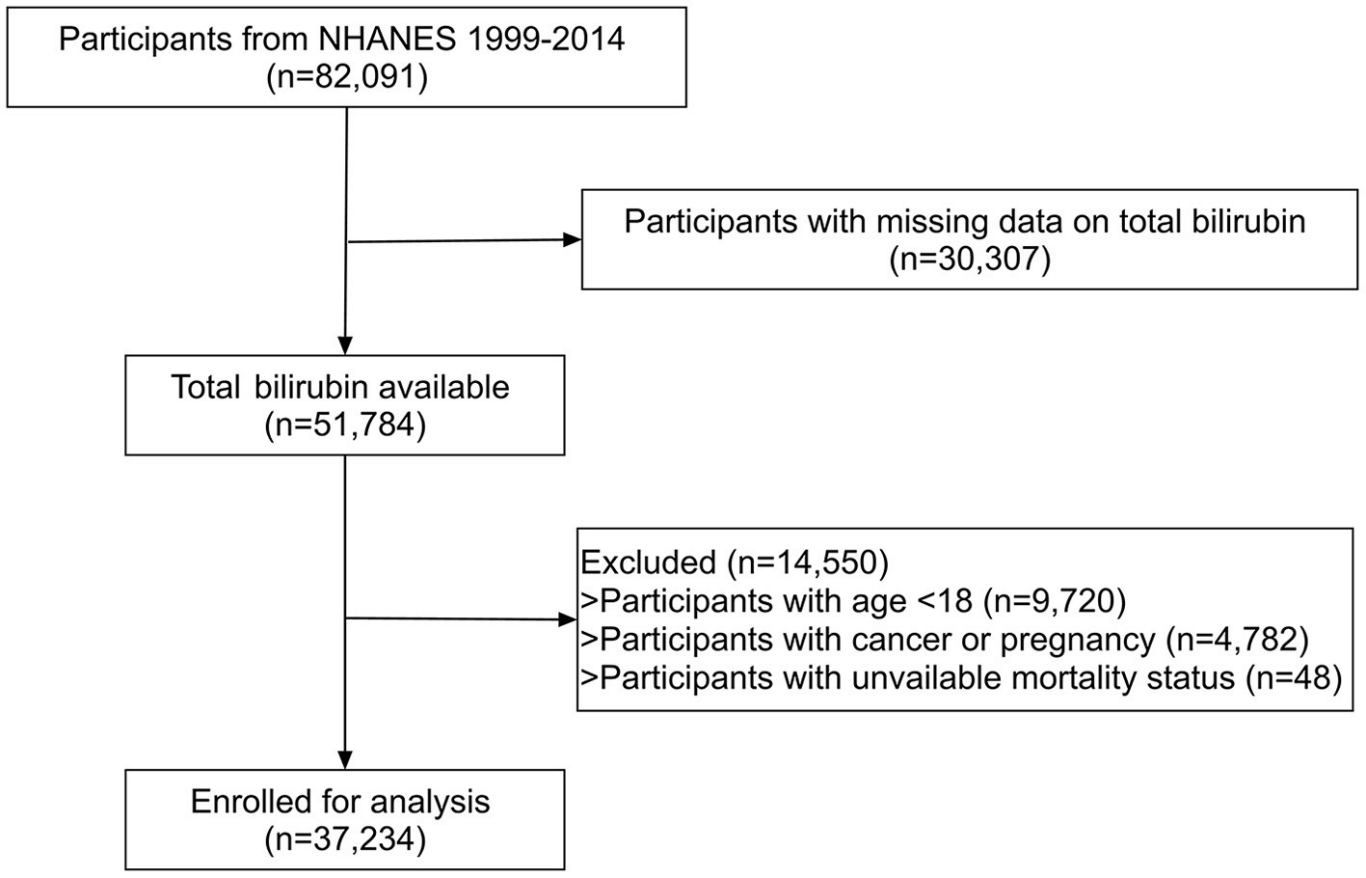
The flow chart of participant selection.

The study included individuals from the National Health and Nutrition Examination Survey (NHANES) between the period of 1999–2014, a nationwide cross-sectional survey conducted by the Center for Disease Control and Prevention (CDC) in the United States (16). After excluding participants with missing total bilirubin data (*n* = 30,307) at baseline, 51,784 participants were available. Participants with age <18 (*n* = 9,720), cancer or pregnancy (*n* = 4,780), and unavailable mortality status (*n* = 48) were further excluded. Finally, 37,234 participants were enrolled in our study. Figure 1 depicted the selection process. All participants provided written informed consent and the protocol was approved by NCHS Research Ethics Review Board (Protocol #98-12, Protocol #2005–06, and Protocol #2011–17).

### Endpoints

The primary outcome was all-cause mortality while the secondary outcomes included death from cardiac-specific and cancer-specific disease. Mortality status was obtained by linkage to the National Death Index by December 31, 2015. Cardiac-specific disease was defined as ICD-10 codes I00-I09, I11, I13, or I20-I51. Cancer-specific disease was defined as ICD-10 codes C00-C97.

### Covariates' Collection

Serum bilirubin levels were measured using a timed-endpoint Diazo method across all individuals. Briefly, bilirubin reacts with diazo reagent in the presence of caffeine, benzoate, and acetate as accelerators to form azobilirubin. The system monitors the change in absorbance at 520 nm at a fixed-time interval. This change in absorbance is directly proportional to the concentration of total bilirubin in the sample.

Information on age, gender, race, education, poverty income ratio (PIR), drinking, smoking, physical activity, medical history (hypertension, diabetes, and cardiovascular diseases), medications (antihypertensive drugs, hypoglycemic drugs, and lipid-lowering drugs) were available. Alanine aminotransferase (ALT), aspartate aminotransferase (AST), albumin, cholesterol, triglycerides, high density lipoprotein (HDL), low density lipoprotein (LDL), and C-reactive protein (CRP) were obtained and measured by standard biochemistry assays. ALT and AST were measured using an enzymatic rate method. Albumin was measured using a biochromatic digital endpoint method. Cholesterol was measured using a timed-endpoint method. Triglycerides and LDL were measured enzymatically using Roche Modular P chemistry analyzer. HDL was measured using a direct immunoassay method. CRP was measured using a latex-enhanced nephelometry method. The details of laboratory methodology are available at https://wwwn.cdc.gov/Nchs/Nhanes/2013-2014/BIOPRO_H.htm. Body mass index (BMI) was calculated as weight in kilograms divided by the square of height in meters. Race was classified as non-Hispanic white, non-Hispanic black, Mexican American, other Hispanic, or others. Education level was categorized as less than high school, high school, college or equivalent, or above. Poverty income ratio (PIR) was stratified as <1, 1–3, and>3. Smoking status was defined as current, past, and never. Physical activity status was classified as vigorous, moderate, and inactive. The estimated glomerular filtration rate (eGFR) was calculated according to the Chronic Kidney Disease-Epidemiology Collaboration (CKD-EPI) equation (17). Hypertension was defined as a history of hypertension or blood pressure ≥140/90 mm Hg or taking anti-hypertensive medications. Diabetes was defined as a history of diabetes or fasting glucose >7 mmol/L or glycated hemoglobin A_1c_ >6.5% or use of hypoglycemic medication. Cardiovascular disease (CVD) was defined as self-reported congestive heart failure, coronary heart disease, angina pectoris, heart attack, and stroke (18). The participant was recorded as having CVD if she/he answered “yes” to the following question: “Has a doctor or other health professional ever told you that you had congestive heart failure/coronary heart disease/angina pectoris/stroke?” in a validated questionnaire (https://wwwn.cdc.gov/Nchs/Nhanes/2013-2014/MCQ_H.htm). Multiple imputation using predictive mean matching (PMM) was performed for covariates with missing values.

### Statistical Analysis

Descriptive statistics was presented according to the levels of bilirubin (Q1: ≤ 8.55 μmol/L; Q2: 8.55~11.97 μmol/L; Q3: 11.97~13.68 μmol/L; Q4: >13.68 μmol/L) and group differences were explored by one-way analysis of variance and chi-square tests. Associations between levels of bilirubin and the risk of all-cause and specific-cause mortality were estimated by multivariate Cox regression models with 95% confidence intervals. Model 1 was adjusted for age and gender. Model 2 was adjusted for age, gender, education level, race, PIR, BMI, smoker, drinking, physical activity, hypertension, diabetes, anti-hypertensive drug, hypoglycemic drug, and lipid-lowering drug. Model 3 was adjusted for age, gender, education level, race, PIR, BMI, smoker, drinker, physical activity, hypertension, diabetes, CVD, anti-hypertensive drug, hypoglycemic drug, lipid-lowering drug, ALT, AST, cholesterol, triglycerides, HDL, LDL, CRP, and eGFR. The associations between levels of bilirubin and all endpoints were evaluated on a continuous scale with restricted cubic spline curve with 3 knots (0.10, 0.50, and 0.90, respectively) based on the Cox proportional hazards models. The critical point of the bilirubin was chosen based on the hazard ratio equal to one. Subgroup analyses were conducted to examine whether the investigated associations between bilirubin and all-cause mortality were modified by age, gender, diabetes, hypertension, and cardiovascular diseases based on the fully adjusted multivariable regression model with interactions between bilirubin and stratified covariates. R software version 3.6.0 was used for all the statistical analyses, and a *p* < 0.05 was considered significant.

## Results

The baseline characteristics of the study population according to bilirubin quartiles were shown in Table 1. The highest quartile tended to be older, male, and had increased levels of ALT and AST. However, triglycerides decreased with increasing bilirubin concentration. The lowest bilirubin concentration had a higher percentage of hypertension and diabetes while a lower percentage of CVD.

**Table 1:** Characteristics of the study population.

**Variable**	**Q1 (*n* = 9,963)**	**Q2 (*n* = 7,949)**	**Q3 (*n* = 10,153)**	**Q4 (*n* = 9,169)**	***P*-value**
Total bilirubin, μmol/L	7.24 (1.49)	10.09 (0.51)	12.70 (0.84)	19.03 (6.25)	<0.001
Male (%)	3,073 (30.8)	3,569 (44.9)	5,592 (55.1)	6,498 (70.9)	<0.001
Age, years	45.11 (18.16)	46.80 (18.89)	47.11 (18.92)	45.57 (19.47)	<0.001
Race (%)		<0.001
Non-Hispanic white	3,734 (37.5)	3,352 (42.2)	4,645 (45.8)	4,428 (48.3)	
Non-Hispanic black	2,595 (26.0)	1,766 (22.2)	1,986 (19.6)	1,613 (17.6)	
Mexican American	2,013 (20.2)	1,748 (22.0)	1,987 (19.6)	1,727 (18.8)	
Others	1,621 (16.3)	1,083 (13.6)	1,535 (15.1)	1,401 (15.3)	
Education (%)		<0.001
Less than high school	2,886 (31.7)	2,331 (31.8)	2,613 (27.7)	2,043 (24.7)	
High school or equivalent	2,186 (24.0)	1,719 (23.4)	2,141 (22.7)	1,893 (22.8)	
College or above	4,045 (44.4)	3,282 (44.8)	4,666 (49.5)	4,350 (52.5)	
PIR (%)		<0.001
<1	2,487 (27.3)	1,659 (22.9)	1,955 (20.9)	1,671 (19.7)	
1~3	3,856 (42.3)	3,076 (42.4)	3,841 (41.1)	3,342 (39.4)	
>3	2,783 (30.5)	2,515 (34.7)	3,556 (38.0)	3,468 (40.9)	
BMI, kg/m^2^	29.74 (7.48)	28.85 (6.74)	28.21 (6.40)	27.22 (5.70)	<0.001
Drinking (%)	1,453 (49.0)	1,106 (52.2)	1,209 (49.7)	890 (49.0)	0.103
Smoking (%)		<0.001
Current	2,087 (28.2)	1,512 (26.5)	1,609 (22.5)	1,120 (18.4)	
Past	359 (4.9)	307 (5.4)	373 (5.2)	341 (5.6)	
Never	4,943 (66.9)	3,890 (68.1)	5,165 (72.3)	4,634 (76.0)	
Activity (%)		<0.001
Vigorous	2,066 (38.0)	1,970 (43.2)	2,614 (42.9)	2,749 (47.0)	
Moderate	2,262 (41.6)	1,842 (40.4)	2,419 (39.7)	2,098 (35.8)	
Inactive	1,103 (20.3)	749 (16.4)	1,056 (17.3)	1,006 (17.2)	
Past history (%)
Hypertension	1,803 (18.9)	1,515 (19.9)	1,776 (18.3)	1,536 (17.5)	0.001
Diabetes	1,582 (15.9)	1,139 (14.3)	1,321 (13.0)	1,071 (11.7)	<0.001
CVD	665 (7.3)	593 (8.1)	716 (7.6)	725 (8.7)	0.002
Prior medication (%)
Antihypertensive drug	2,588 (83.2)	2,077 (83.8)	2,593 (82.8)	2,102 (82.9)	0.772
Hypoglycemic drug	831 (54.5)	558 (56.0)	687 (54.2)	500 (51.0)	0.144
Lipid-lowering drug	1,382 (80.8)	1,067 (78.2)	1,484 (79.9)	1,156 (79.5)	0.339
ALT, IU/L	23.10 (15.81)	24.79 (17.42)	26.45 (27.81)	28.42 (36.49)	<0.001
AST, IU/L	23.75 (13.83)	24.67 (13.33)	26.24 (21.28)	28.25 (26.21)	<0.001
Albumin, g/L	4.17 (0.34)	4.27 (0.33)	4.30 (0.31)	4.40 (0.33)	<0.001
Cholesterol, mg/dL	191.4 (40.4)	195.1 (41.5)	196.4 (42.9)	192.5 (43.3)	<0.001
Triglycerides, mg/dL	154.3 (124.0)	149.1 (118.3)	146.0 (137.9)	138.0 (146.8)	<0.001
HDL, mg/dL	51.5 (15.0)	51.8 (15.5)	52.8 (15.7)	52.3 (15.5)	<0.001
LDL, mg/dL	112.3 (34.7)	116.7 (36.1)	115.9 (35.9)	114.2 (35.9)	<0.001
CRP, mg/dL	0.59 (1.02)	0.46 (0.84)	0.37 (0.66)	0.32 (0.87)	<0.001
eGFR, ml/min per 1.73 m^2^	98.97 (32.16)	97.44 (30.46)	92.92 (30.04)	94.52 (26.80)	<0.001

*Data are presented as mean (SD) or n (%). Q1: total bilirubin ≤ 8.55 μmol/L; Q2: 8.55~11.97 μmol/L; Q3: 11.97~13.68 μmol/L; Q4: total bilirubin>13.68 μmol/L. PIR, poverty income ratio; BMI, body mass index; CVD, cardiovascular diseases; ALT, alanine aminotransferase; AST, aspartate aminotransferase; HDL, high density lipoprotein; LDL, low density lipoprotein; CRP, C-reactive protein; eGFR, estimated glomerular filtration rate*.

As shown in Table 2, after adjusting for demographics, lifestyles, medical history, and biochemical index, total bilirubin was positively associated with all-cause mortality [HR 1.59, 95% CI (1.46–1.72), *p* < 0.001]. When compared with the lowest quartile of participants (total bilirubin ≤ 8.55 μmol/L), the multivariable adjusted HR for all-cause mortality was 1.25 [95% CI (1.14, 1.37), *p* < 0.001] for individuals with total bilirubin>13.68 μmol/L. Also, an increased risk of cardiovascular mortality [HR 1.53, 95% CI (1.26, 1.85), *p* < 0.001] and cancer mortality [HR 1.44, 95%CI (1.20, 1.74), *p* < 0.001] were also seen at higher bilirubin levels.

**Table 2:** Association of total bilirubin with all-cause and cause-specific mortality.

**All cause and cause specific mortality**	**Cases**	***N***	**Model 1 HR(95% CI)**	**Model 2 HR(95% CI)**	**Model 3 HR(95% CI)**
**All causes**
Q1	965	9,963	Ref	Ref	Ref
Q2	985	7,949	0.81 [0.74, 0.89]^***^	0.87 [0.80, 0.96]^**^	0.87 [0.80, 0.95]^**^
Q3	1,011	10,153	1.18 [1.08, 1.29]^***^	1.35 [1.23, 1.48]^***^	1.26 [1.15, 1.38]^**^
Q4	1,048	9,169	1.13 [1.03, 1.24]^**^	1.32 [1.20, 1.45]^***^	1.25 [1.14, 1.37]^***^
Continuous	4,009	37,234	1.49 [1.38, 1.62]^***^	1.71 [1.58, 1.86]^***^	1.59 [1.46, 1.72]^***^
**Cardiovascular**
Q1	155	962	Ref	Ref	Ref
Q2	183	981	0.95 [0.76, 1.18]	1.00 [0.81, 1.25]	0.99 [0.80, 1.23]
Q3	189	1,010	1.54 [1.24, 1.92]^***^	1.67 [1.34, 2.09]^***^	1.56 [1.24, 1.95]^***^
Q4	185	1,047	1.28 [1.03, 1.60]^*^	1.42 [1.13, 1.78]^**^	1.36 [1.08, 1.71]^**^
Continuous	712	4,000	1.49 [1.24, 1.78]^***^	1.60 [1.33, 1.93]^***^	1.53 [1.26, 1.85]^***^
**Malignant neoplasms**
Q1	189	962	Ref	Ref	Ref
Q2	183	981	0.85 [0.69, 1.04]	0.84 [0.68, 1.03]	0.82 [0.67, 1.01]
Q3	195	1,010	1.41 [1.15, 1.73]^**^	1.36 [1.11, 1.68]^**^	1.27 [1.03, 1.57]^*^
Q4	189	1,047	1.17 [0.95, 1.44]	1.18 [0.96, 1.47]	1.10 [0.89, 1.37]
Continuous	756	4,000	1.52 [1.28, 1.81]^***^	1.55 [1.30, 1.85]^***^	1.44 [1.20, 1.74]^***^

*Model 1 was adjusted for age and gender*.

*Model 2 was adjusted for age, gender, education level, race, PIR, BMI, smoker, drinker, physical activity, hypertension, diabetes, CVD, anti-hypertensive drug, hypoglycemic drug, lipid-lowering drug*.

*Model 3 was adjusted for age, gender, education level, race, PIR, BMI, smoker, drinker, physical activity, hypertension, diabetes, CVD, anti-hypertensive drug, hypoglycemic drug, lipid-lowering drug, ALT, AST, cholesterol, triglycerides, HDL, LDL, CRP, eGFR*.

* *p < 0.05,*

** *p < 0.01,*

*** *p < 0*.

**Figure 2:**
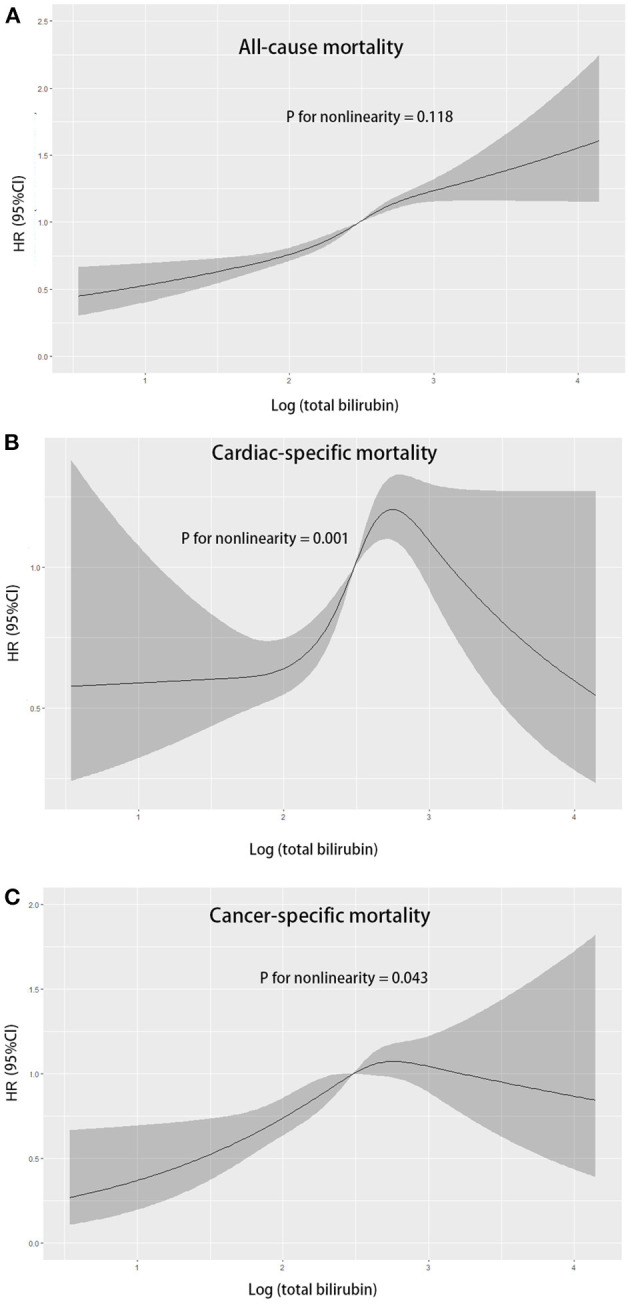
Multivariable adjusted hazard ratios for all-cause mortality **(A)**, cardiovascular mortality **(B)**, and cancer mortality **(C)** according to levels of total bilirubin levels on a continuous scale (log-transformed) in overall population. Solid blue lines are multivariable adjusted hazard ratios, with shadow lines showing 95% confidence intervals derived from restricted cubic spline regressions.

A non-linear relationship was explored using restricted cubic splines regression. Total bilirubin levels were linearly associated with the higher risk of all-cause mortality (Figure 2A; *p* for non-linearity = 0.118). When the bilirubin concentration was <12.2 μmol/L (log12.2 = 2.5), the bilirubin showed a protective role. The association between bilirubin levels and the risk of cardiovascular and cancer mortality was non-linear. A range of 12.2–18.0 μmol/L was significantly associated with a high risk of cardiovascular mortality, while higher bilirubin levels were not (Figure 2B). However, the 95% confidence interval included the hazard ratio of 1.0 above the concentration of 12.2 μmol/L for cancer mortality (Figure 2C).

To observe significant interactions for association between total bilirubin with all-cause mortality, we stratified the individuals by age, gender, hypertension, diabetes, and cardiovascular diseases. Subgroup analysis (Table 3) showed that the association of total bilirubin with all-cause mortality was consistent across gender, age, high blood pressure, diabetes, and cardiovascular diseases groups. The association was more pronounced in individuals aged 65 or above [HR 1.66, 95% CI (1.49, 1.84), *p* for interaction = 0.045] or hypertension populations [HR 1.96, 95% CI (1.71, 2.24), *p* for interaction = 0.016].

**Table 3:** Subgroup analysis of association of total bilirubin with all-cause mortality.

	**HR (95%CI)**	***P* for interaction**
Gender		0.967
Female	1.60 [1.40, 1.82]^***^	
Male	1.57 [1.40, 1.75]^***^	
Age		0.045
<65	1.37 [1.19, 1.58]^***^	
≥65	1.66 [1.49, 1.84]^***^	
Diabetes		0.271
No	1.52 [1.38, 1.68]^***^	
Yes	1.73 [1.48, 2.02]^***^	
CVD		0.072
No	1.49 [1.35, 1.64]^***^	
Yes	1.92 [1.61, 2.27]^***^	
Hypertension
No	1.37 [1.23, 1.53]^***^	0.016
Yes	1.96 [1.71, 2.24]^***^	

*** *p < 0.001*.

## Discussion

In this large prospective study of 37,234 adults recruited from a nationally representative sample of the United States, our findings demonstrated that higher levels of bilirubin were independently associated with increased all-cause and cause-specific mortality. Such association remained significant across subgroups. Restricted cubic splines indicated that the association was non-linear in cardiovascular mortality and cancer mortality while it was linear in all-cause mortality.

Bilirubin is the end product of heme catabolism and has antioxidant and anti-inflammatory effects (19). However, our study found total bilirubin levels increased the risk of all-cause mortality. This is consistent with other previous studies (15, 20). A high risk of cardiovascular disease could contribute to increased mortality. In addition, subgroup analysis confirmed that a history of diabetes, cardiovascular diseases, and high blood pressure increased the risk of total bilirubin for all-cause mortality. In this regard, previous studies found that total bilirubin levels are positively associated with the severity of CAD in patients with STEMI or non-STEMI (21, 22).

However, several studies investigating the relation between levels of plasma bilirubin and the risk of all-cause mortality have found inverse association. HAPIEE cohort had supported the negative relationship between bilirubin and total mortality and cancer mortality (23). In a male Belgian population, high serum bilirubin within normal ranges was associated with lower all-cause and cancer mortality (24). A study that enrolled older adults from NHANES found that the association of total bilirubin levels with total mortality was the highest among those with a level between 0.1 and 0.4 mg/dl and it could not investigate whether higher total bilirubin levels were associated with a higher mortality risk (25). Our study had a large sample size of US adults with more representative bilirubin levels and our positive correlation remained consistent across gender. A large cohort study in the UK found a per 0.1-mg/dl increase in bilirubin level indicated a 3% decrease for mortality (26), but the bilirubin levels were lower compared with our study. A negative relationship was also found in particular populations, such as chronic hemodialysis patients (27) and diabetic patients (8). Some diseases could lead to the metabolism change of total bilirubin, thereby influencing the association.

Some possible explanations could exist. Firstly, a high level of bilirubin could be a reflection of increased oxidative stress and chronic inflammation response, which predicted poor prognosis. Secondly, high bilirubin levels cause cell toxicity and tissue injury. Thirdly, bilirubin was elevated in liver diseases, which contributed to the association with higher mortality risk. Finally, circulating bilirubin, as a surrogate biomarker of cardiac dysfunction and poor hepatic perfusion, could exert an effect on the blood pressure and other cardiovascular risk (28). However, these speculations require further investigation.

Our study has some limitations. First, some covariates were self-reported using validated questionnaires. Second, data on total bilirubin was only collected once at baseline, and it was unclear whether bilirubin changes over time could affect the association with mortality.

## Conclusions

In summary, we found that high bilirubin levels were associated with an increased risk of all-cause, cardiovascular, and cancer mortality in US adults. It is favorable to control total bilirubin under 12.2 μmol/L.

## Data Availability Statement

The original contributions generated for the study are included in the article/supplementary materials, further inquiries can be directed to the corresponding author/s.

## Ethics Statement

The studies involving human participants were reviewed and approved by National Center for Health Statistics. The patients/participants provided their written informed consent to participate in this study.

## Author Contributions

QL designed the study. ZC wrote the main manuscript. JH and CC prepared the figures and tables. All authors contributed to the article and approved the submitted version.

## Conflict of Interest

The authors declare that the research was conducted in the absence of any commercial or financial relationships that could be construed as a potential conflict of interest.
